# Editorial: New molecular approaches to improve gynecological cancer management

**DOI:** 10.3389/fonc.2023.1235035

**Published:** 2023-06-21

**Authors:** Miguel Henriques Abreu, Gabriella Lillsunde-Larsson, Carla Bartosch, Sara Ricardo

**Affiliations:** ^1^ Department of Medical Oncology, Portuguese Oncology Institute of Porto (IPO-Porto), Porto, Portugal; ^2^ Porto Comprehensive Cancer Center Raquel Seruca (PCCC), Porto, Portugal; ^3^ Department of Laboratory Medicine, Faculty of Medicine and Health, Örebro University, Örebro, Sweden; ^4^ School of Health Sciences, Örebro University, Örebro, Sweden; ^5^ Department of Pathology, Portuguese Oncology Institute of Porto (IPO-Porto), Porto, Portugal; ^6^ Cancer Biology & Epigenetics Group, Research Center of Portuguese Oncology Institute of Porto (CI-IPO-Porto)/Health Research Network (RISE@CI-IPO-Porto), Portuguese Oncology Institute of Porto (IPO-Porto), Porto, Portugal; ^7^ Differentiation and Cancer Group, Institute for Research and Innovation in Health (i3S) of the University of Porto, Porto, Portugal; ^8^ 1H-TOXRUN – One Health Toxicology Research Unit, University Institute of Health Sciences (IUCS), CESPU, CRL, Gandra, Portugal; ^9^ Department of Pathology, Faculty of Medicine from University of Porto (FMUP), Porto, Portugal

**Keywords:** gynecology oncology, cervical cancer, ovarian cancer, endometrial cancer, vaginal cancer, vulvar cancer

Gynecological cancers are major contributors to women cancer burden. Deaths estimatives for 2040, stratified by countries income level (**Figure 1**), unveils the lack of access to early diagnosis and inequalities in management in countries with low Human Development Index (HDI). In these countries, the average risk of death by gynecological cancers in 2040 is 100% whereas in very high HDI countries the risk raises in average 33%. This estimatives illustrates an accentuation of discrepancies related to the risk of death from gynecological cancer. In countries with less economic resources, cervical cancer continues to be the major cause of death, whereas in very high HDI countries ovarian and endometrial cancer are the ones that contribute most to the mortality index. Developed countries have the responsibility to promote and make available the treatments already implemented that will make the difference for patients’ survival.

**Figure 1:**
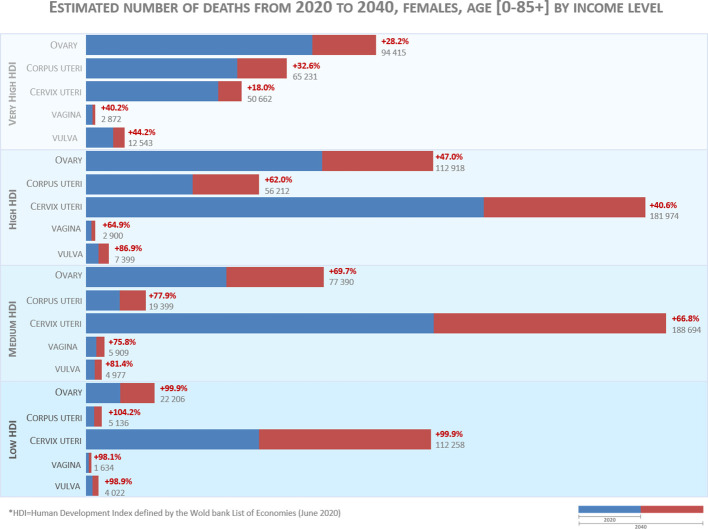
Estimated number of deaths from 2020 to 2040. The data source for graphic construction is Ferlay J, Laversanne M, Ervik M, Lam F, Colombet M, Mery L, Piñeros M, Znaor A, Soerjomataram I, Bray F (2020). Global Cancer Observatory: Cancer Tomorrow. Lyon, France: International Agency for Research on Cancer. Data available from: https://gco.iarc.fr/tomorrow, accessed on May 31^th^ 2023.

In very-high HDI countries, ovarian cancer is the most common cause of gynecological cancer death, the epithelial type being the most frequent. The maximization of the success of the treatment requires expert multidisciplinary care provided in specialised institutions. In these dedicated hospitals, epithelial ovarian cancer patients’ therapies are nowadays guided, at least in part, by genomic tests, such as detection of mutation in BRCA1/BRCA2 and homologous recombination deficiency genes, which improve effectiveness of therapy (1). In this Research Topic, Abbas-Aghababazadeh et al. identified two markers, ADRB2 and FAP, that were associated with increased odds of optimal debulking. Liang et al. proposes a signature based on adenosine metabolism related genes that could be used as a prognostic biomarker to stratify ovarian cancer patients and Roering et al. demonstrated that Wee1 inhibition can affects several critical functions related to proliferation, cell cycle and division, apoptosis and invasion.

Endometrial Cancer is the second cause of death for gynecological cancer in very high HDI countries but the estimative of deaths are raising in less developed economies. One of the main factors contributing to this trend is obesity. Despite the prevalence of obesity, management of endometrial cancer in very-high HDI countries have the advantage of having well-equipped health services, and most endometrial cancers can be cured by surgery with modern techniques of intraoperative staging. Advances in our understanding of the molecular biology of endometrial cancer have changed the way that we stratify the patients in risk groups for relapse and, because of that, altered the definition of adjuvant therapies (2). However, new biomarkers are still needed to further stratify patients, particularly within non-specific molecular profile group. Regarding this point, Parrish et al. made an elegant review dissecting the role of mutant b-catenin in endometrial cancer progression, describing how its functions may change and drive endometrial cancer progression in CTNNB1 mutant patients.

The deaths estimatives for 2040 show that cervical cancer will be the biggest contributor for gynecological cancers deaths. High-risk subtypes of human papilomavirus (HPV) are the main cause of the disease making this type of gynecological cancer preventable. It is therefore alarming that this available preventive method of vaccination is still far from being implemented in countries with lower incomes. Decrease in cervical cancer mortality is a powerful example of how investment in research translates into gains in terms of patient survival. In very high HDI countries, the mortality risk in 2040 will increase 18% mainly because of metastatic or recurrent disease in which the overall prognosis remains poor. Nevertheless, the incorporation of the anti-VEGF agent bevacizumab (3), and most recent immunotherapy agents like pembrolizumab in 1^st^ line with/without bevacizumab and cemiplimab in 2^nd^ line had extend patients overall survival. In this Research Topic, Zou et al. explored the value of miR-326 as a predictive biomarker for response to neoadjuvant chemotherapy in locally advanced cervical cancer. Another promising auxiliary marker was depicted by Li et al. that found that PAX1 methylation status is highly suggestive of invasive cervical cancer and could be useful before conization clinical decision. Finally, Rai et al. elegantly presented a distinct mechanism of cervical cancer cell death caused by Drug SHetA2.

Primary vaginal cancer is rare, representing only 10% of all vaginal malignant neoplasms and only 1–2% of all gynecological cancers (4). Vaginal cancer, like cervical cancer, is strongly associated with the HPV (4) infection. In this section, Shi et al. performed a longitudinal study where it was observed a potential role of vaginal microbiota in the persistent high-risk HPV infections.

Vulvar squamous cell cancer accounts for 90% of vulvar cancers. Next-generation sequencing studies of Vulvar squamous cell cancer imply human papillomavirus and p53 status play separate roles in carcinogenesis and prognosis (5). As many as 40% of patients with Vulvar squamous cell cancer who are initially managed surgically will have a recurrence, which is often fatal. Patients who are not candidates for locoregional treatments, have poor overall survival (5). Non epithelial tumours of the vulva are rare and encompassed several histological types. Kong et al. reported a very rare case of a patient with recurrent vulvovaginal paraganglioma with SDHB gene mutation and review of the literature of a recurrent paraganglioma of the vulva.

Gestational trophoblastic disease encompasses a range of pregnancy-related disorders, consisting of premalignant disorders including complete and partial hydatidiform mole, and malignant disorders such as invasive mole and choriocarcinoma (6). Patients’ management has long been based on FIGO risk score stratification, but additional studies to further improve management have been limited by the rarity of these tumours. In this regard, Wang et al. studied the serum peptide signatures of 65 gestational trophoblastic neoplasia patients and, in combination with FIGO risk score, they showed its potential to predict outcome in these patients after first-line chemotherapy.

Despite current knowledge of the molecular and genetic events behind gynecological cancers, the field still has a lot to improve. Development of new molecular approaches is essential to early detect and treat in a personalised way these diseases. Another important challenge is to guarantee equal access to gynecological cancer treatments. The concept of global health was evidenced in the recent COVID19 pandemic and will have to be a priority to reduce cancer mortality rate worldwide.

## Author contributions

All the authors contributed to the conception and revision of the editorial. SR performed the data analysis and graphical design of **Figure 1**. All authors contributed to the article and approved the submitted version.
